# Epithelial-mesenchymal dynamics in cancer: Role of signalling pathways, stromal interactions and natural therapies

**DOI:** 10.5599/admet.2836

**Published:** 2025-09-08

**Authors:** Iram Khan, Anindita Behera, Kamesh R. Babu, Faisal Rehman, Farah Rehan, Shraddha M. Gupta, Siddharth Singh

**Affiliations:** 1 Department of Pharmaceutical Sciences, School of Health Sciences and Technology, UPES, Dehradun, Uttarakhand, India; 2 Amity Institute of Pharmacy, Amity University, Kolkata, India; 3 Department of Allied Health Sciences, School of Health Sciences and Technology, UPES, Dehradun, Uttarakhand, India; 4 University College Cork, Dublin, Ireland; 5 Department of Molecular Medicine and Al-Jawhara Centre for Molecular Medicine, College of Medicine and Medical Sciences, Arabian Gulf University, Manama, Kingdom of Bahrain; 6 School of Pharmaceutical & Population Health Informatics, DIT University, Dehradun, Uttarakhand, India

**Keywords:** Epithelial-mesenchymal transition, metastasis, drug resistance, therapeutic molecules, oncogenesis

## Abstract

**Background and purpose:**

Epithelial-mesenchymal transition (EMT) and its reverse process, mesenchymal-epithelial transition (MET), play crucial roles in embryogenesis, tissue regeneration, and cancer progression. Dysregulation of EMT/MET pathways in cancer contributes to metastasis and drug resistance.

**Approach:**

This review discusses the signalling pathways that are correlated with EMT in cancer and investigates the therapeutic potential of naturally occurring compounds in modulating these processes. The intricate relationship between stromal cells, drug resistance, and EMT is discussed, highlighting the emerging role of MET in stabilizing distant metastasis. Additionally, the impact of p53 on EMT and its implications in cancer metastasis are discussed. The review also provides an overview of therapeutic molecules, both plant- and animal-derived, that regulate EMT, highlighting their potential in cancer treatment. Specifically, plant-based compounds from *Atractylodes lancea*, *Dendrobium officinale*, *Panax ginseng* and *Platycodon grandiflorus*, as well as animal-derived substances like bee venom and snake venom, are highlighted. Furthermore, marine-based compounds, including caprolactin C, laminaran sulfate, BFP-3, bryostatin 1, sinulariolide, manzamines, halichondrin B, eribulin and biemamides, exhibit significant anti-metastatic effects by targeting EMT-associated pathways.

**Conclusion:**

The diverse range of therapeutic molecules discussed in this review provides promising therapeutic avenues for developing targeted strategies against EMT in cancer.

## Introduction

Epithelial cells are polarized along the apical-basal axis and connected with adjacent cells through intercellular adhesion complexes, including adherens junctions, tight junctions, desmosomes, and gap junctions. These junctions carry different morphological attributes, compositions, and functions. In contrast, mesenchymal cells are non-polarized, and the intercellular adhesion complexes are downregulated and mislocated, which favours the mesenchymal cell mobility through the extracellular matrix (ECM) [1].

Physiologically, the epithelial and mesenchymal cells can be transformed into each other, and the event is called EMT (epithelial to mesenchymal transition) and MET (mesenchymal to epithelial transition). Interestingly, both MET and EMT are reversible processes and are extensively observed during normal physiological conditions, including embryogenesis and organogenesis [2].

EMT is a natural process that transpires within embryonic development, tissue regeneration, and also in cancer progression [2-4]. The transformed mesenchymal phenotype cells exhibit elevated migration, invasion, apoptosis resistance, and ECM components formation. EMT phenomenon can be classified into three types based on their origin: type 1 is associated with gastrulation and organ development; type 2 promotes tissue regeneration and wound repair. Furthermore, type 3 is correlated with the progression of cancer, encompassing the mechanisms by which tumour cells invade, migrate, and metastasize [3]. MET plays a pivotal role in embryogenesis in assembling non-polarized mesenchymal-like cells into connected cell structures [2]. During the process of MET, genes associated with the mesenchymal phenotype are downregulated, while those associated with the epithelial phenotype are upregulated. Furthermore, studies have demonstrated that MET exerts a substantial influence on the mechanisms underlying metabolic switch and epigenetic modifications [5].

Both EMT and MET are strictly regulated processes, but these processes are highly dysregulated in cancer to favour tumour progression by metastasis. The role of MET is little known in cancer, whereas the significance of EMT is well-studied in tumour progression. The broadest range of tumours undergo EMT to promote early-stage tumour cells to gain infiltrating and metastasizing characteristics during tumour progression [6]. However, benign tumour cells of carcinosarcoma maintain both epithelial and mesenchymal characteristics and exhibit high malignancy [6]. Numerous investigations have shown that the initiation of EMT in cancer cells might result in the production of cancer stem cells (CSCs). The EMT-transformed epithelial cancer cells exhibit stem-like characteristics that favour the generation of primary tumours, increased metastasis, and the outgrowth of tumours at distant organs [7]. Interestingly, MET is involved in the organization and stabilization of distant metastasis by promoting mesenchymal-like cancer cells to attain epithelial-like characteristics and incorporate them into distant organs [6].

In recent years, researchers have begun to investigate the EMT and MET pathways as potential targets for preventing metastasis in cancer [8,9]. Many naturally occurring compounds are being investigated for their potential as potent modulators of EMT-related pathways. These compounds modulate the EMT by regulating genes associated with the EMT process and inhibit cancer progression [10,11]. In this review, we discuss the signalling pathways and genes associated with EMT in cancer progression, as well as the role of naturally occurring therapeutic compounds that inhibit cancer progression by modulating the EMT process.

## Epithelial-mesenchymal transition-related signalling pathways

The epithelial-mesenchymal transition involves several biological and biochemical alterations, which are regulated by different signalling meta pathways. The SNAIL, TWIST, and ZEB are the transcription factors (TFs) that suppress epithelial cell characteristics and activate the genes of mesenchymal cells [12]. The signalling pathways can induce the EMT alone, but they also intermingle with other TFs and produce a complicated nonlinear EMT signalling network [13]. SNAIL1 and SNAIL2 are the important SNAIL proteins that participate in all types of EMT. These SNAIL proteins with polycomb repressive complex 2 (PCR2) suppress the expression of epithelial genes like occludins, E-cadherin, cadherin-16, and transcription factor 2 (TCF2), which promotes the detachment of epithelial cells and enhances the expression of mesenchymal genes like ZEB1 and N-cadherin and extracellular matrix metalloproteinases (MMP9 and MMP2), promoting the invasion of mesenchymal phenotypes [14,15]. ZEB or zinc finger E-box binding proteins can activate or suppress various epithelial and mesenchymal proteins to induce EMT [16]. ZEB1 and ZEB2 activate and cause over-expression of epithelial markers like cadherins, proteins of epithelial gaps and polarity proteins, and mesenchymal genes like N-N-cadherins and vimentin simultaneously. ZEB1 is also regulated directly by Wnt and Notch signalling pathways [17,18]. TWIST1 and TWIST2 are β-helix-loop-helix proteins that restrict the transcription of DNA in epithelial genes and trigger mesenchymal gene expression. Many signalling pathways activate TWIST proteins [15].

Other than these, countless *in vitro* and *in vivo* experiments have reported the involvement of various pathways in EMT. In EMT, several pathways are involved. These include tyrosine kinase inhibitors, hepatocyte growth factor, epidermal growth factor, fibroblast growth factor, platelet-derived growth factor, insulin-like growth factors, Wnt/β-catenin, nuclear factor kappa B, transforming growth factor-β, and integrin pathways [19]. Downregulation of different components of cell junctions is the main event involved in EMT. E-cadherin expression downregulation causes the dissociation of cells and invasion [20]. TWIST, SNAIL1, SNAIL2, E47, and Smad-interacting protein 1 (SIP1)/ZEB are primarily involved in the downregulation of E-cadherin expression [16]. Overexpression of different transcription factors in epithelial cells facilitates the EMT, and downregulation of E-cadherin influences cell migration, proliferation, differentiation, and apoptosis [21].

Inflammatory cytokines in the cancer cells, like interleukin 6, tumour necrosis factor-α, and lipopolysaccharides, induce the nuclear factor kappa B (NF-κB) pathway, which activates the SNAIL and ZEB proteins to activate EMT [22]. NF-κB pathway downregulates E-cadherin and increases the expression of Vimentin, a mesenchymal gene. The NF-κB pathway activates SNAIL, ZEB1, and ZEB2 during the loss of epithelial phenotype, leading to the suppression of E-cadherin and the overexpression of transforming growth factor-β [23]. Wnt/β-catenin upregulates several mesenchymal markers such as TWIST, SLUG, and ZEB1, which suppress E-cadherin and increase the levels of different matrix metalloproteinases like MMP-3, MMP-7 and MMP-9 [24].

## Epithelial-mesenchymal transition role in cancer progression

The EMT plays an important role in different pathological processes and is classified into types I, II, and III. In type I EMT, the epithelial cells are converted into secondary epithelial cells, which are found during embryonic development and organ formation. Type-II EMT leads to the development of fibroblasts from secondary epithelial cells during wound healing, tissue regeneration, and organ fibrosis. Type-III EMT leads to the conversion of primary epithelial cancer cells to metastatic cancer cells, which increases the mobility of secondary epithelial cancer cells, causing cancer progression [24,25]. In some cancer cells, the overexpression of EMT transcription factors drives and increases the incidence of tumourigenicity [26]. Downregulation of E-cadherin promotes the epithelial-mesenchymal transition, facilitating the progression of tumour cells from primary epithelial cancer cells to secondary epithelial cancer cells and ultimately leading to metastasis. Low and high levels of TWIST proteins lead to tumour process initiation and EMT induction, followed by cancer progression, respectively [27].

## Epithelial-mesenchymal transition role in metastasis

EMT, in collaboration with other mechanisms, influences the metastasis of cancer cells [28]. Initially, the population of mesenchymal cells is found to be less than 10 % in xenograft models [29]. Initiation of metastasis is directly supported by EMT in circulating cells. The transcription factors ZEB1 and SLUG are reported to be involved in the metastasis of colorectal cancer and breast cancer [30,31]. The invasion and proliferation of highly metastatic mammary carcinoma cells in the lungs is inhibited by the Downregulation of TWIST proteins [32]. The transcription factors like TWIST1 and SNAI1 facilitate the metastasis of breast cancer to other vital organs [33,34]. Overexpression of TWIST1 is involved in the dissemination of squamous cell cancer; however, once the cells reach the metastatic site, downregulation of TWIST1 facilitates the colonization of metastatic cancer cells [35]. Similarly, metastatic breast cells require the expression of SNAIL for invasion and proliferation; however, once they reach the lung, downregulation of this protein causes the accumulation of metastatic cells [34]. Loss of p120-catenin in EMT promotes the tumour progression and metastasis of pancreatic cancer, but p120-catenin-influenced stabilization of E-cadherin leads to colonization of metastatic pancreatic cells at the liver site [36].

## Epithelial-mesenchymal transition role in drug resistance

For many decades, cancer has ranked second in terms of mortality worldwide. Despite surgical and chemotherapy being the main strategies to cure cancer, some patients develop resistance to chemotherapy [37]. However, the resistance problem to chemotherapy is multifaceted since each tumour has its own defined set of characteristics to favour tumour progression and eventually cause death [38]. We need to take the reductionist approach to define key determinants of drug resistance as well as the different mechanisms through which resistance occurs, including multidrug resistance (MDR), cell death inhibition, alteration in drug metabolism, gene amplification, epigenetic, and drug targets [38,39]. Although over the last decades, advancements in DNA microarray, proteomics technology, and DNA microarray, proteomics technology, and the development of targeted therapies have provided the platform to overcome drug resistance. But still, an effective chemotherapeutic agent that can cure the advanced stage of cancer, such as metastasis and invasion, has not been discovered yet [39,40]. Recent studies have shown that Drug resistance in cancer arises through the process of EMT as a response to existing therapies [41]. Sommers *et al.* [42] proposed that the association between EMT and the development of drug resistance in cancer cells was established in the early 1990s, when they discovered that a vinblastine-resistant ZR-75-B cell line and two Adriamycin-resistant MCF-7 cell lines underwent EMT.

**Figure 1.**
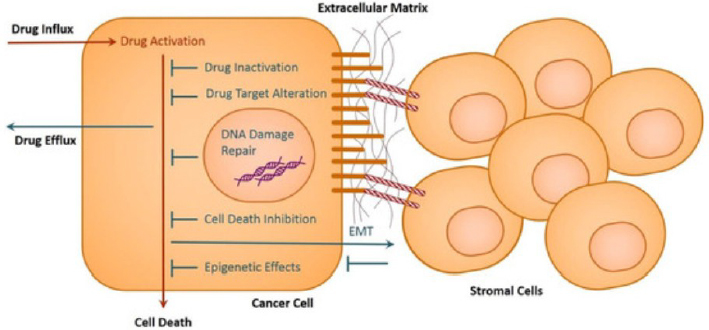
Schematic representation of principal pathways facilitating drug resistance in cancer cells [41] (Creative Commons Attribution CC BY 3.0)

Stromal cells help in EMT and provide signals for enhanced drug resistance in cancer cells. Cancer cells' cell adhesion molecules are adhered to by extracellular matrix proteins and cell adhesion molecules on stromal cells. Factors that control EMT are also secreted by cancer and stromal cells. A condensed example of these cell interactions is illustrated in Figure 1 [41].

Additionally, EMT has been linked to therapy resistance in numerous preclinical models, even though clinical trials and clinical samples have only provided minimal evidence [40]. It has been reported that EMT is one of the mechanisms through which non-small cell lung cancer (NSCLC) patients develop resistance to first-generation reversible EGFR inhibitors gefitinib and erlotinib [43,44].

Still, the connectivity between the cancer metastasis process and EMT has not been completely understood, and perhaps its imprint on drug resistance is becoming more evident. Several EMT pathways can cause drug resistance in cancer cells and are mostly associated with acquired and innate mechanisms of tumour suppressor silencing. In addition, cells experiencing EMT exhibit characteristics that are similar to those of CSC (cancer stem cells). Therefore, the novel emerging therapeutic strategy is a combination of anti-cancer drugs with EMT inhibitors [45-47].

## Mechanism involved during epithelial-mesenchymal transition -induced drug resistance

The exact mechanism involved in the medication resistance and EMT is still unclear [46]. Several factors are supposed to be involved during EMT that play a significant role in drug resistance development. These factors are based on how far the tumour has spread, which defines the differentiation level of EMT. Lesniak et al reported that when ERBB2 (HER2) positive breast cancer is present, tumours with elevated β1 integrin expression become more resistant to antibody inhibitors like trastuzumab [41,42]. As an additional component, differentiation signalling is crucial, which is important for EMT. Because Wnt, Notch, and Hedgehog signalling pathways, and many others, were shared by EMT cells and CSCs. Tumour cells can thus develop resistance to anticancer medications and evade drug-induced cell death thanks to EMT. According to Bates *et al.* and Wang *et al.* [48,49], drug resistance is caused by the increased expression of integrin αvβ1 in colon cancer, which positively influences the expression of transforming growth factor β (TGFβ), which is essential for EMT. In addition, Asiedu et al also confirmed that EMT induced by the TGF-β pathway in breast cancer cells mediates resistance to doxorubicin (DOX) and paclitaxel [50]. Even though EMT promotes the growth of additional metastatic cancer cells, it is also known to send out signals that boost survival, which may lead to medication resistance in part or all the tumour’s cells. To transmit such signals, for example, integrin αvβ1 combines with stromal cell adhesion molecules. Comparably, in mammary cancer, TGFβ-mediated EMT is regulated by β3 integrin and src, but in lung malignancies, integrin β1 ligation triggers proliferative and survival signal-mediated FAK kinase [41]. Another study by Xie *et al.* [51] also reported the highly up-regulated expression of Notch-1 in cancer cells of gefitinib-resistant PC9/AB2 lung due to the Notch-1 receptor intracellular domain (N1IC), the activated form of the Notch-1 receptor, in PC9 cells. Thus, providing the best evidence that gefitinib-acquired resistance in cancer cells of the lung undergoing EMT occurs through Notch-1 signalling activation [47,51]. Increased drug efflux and sluggish cell growth are also components of the general process underlying EMT-associated drug resistance. By altering the expression of molecules involved in immunosuppression or immunoevasion, EMT also plays a significant role in evading immunelogical responses, thereby increasing treatment resistance [25,40,52].

Another important factor contributing to EMT-induced drug resistance is transcription factors that induce metamorphosis (EMT-TFs). These EMT-TFs increase drug efflux via ABC transporters, which in turn promote resistance [7]. Several key signalling pathways, including Notch, TGF-β, Hedgehog, and Wnt are also involved in the expression and regulation of a complex network of EMT-TFs of the ZEB, SNAIL, and TWIST. These EMT-TFs directly block an excess of cell-cell adhesion genes and directly or indirectly also induce the expression of genes involved in cytoskeletal reorganisation, which results in a breakdown of the ECM, and survival of cancer cells, so they can invade and migrate and eventually cause drug resistance [7,53]. Furthermore, ABCB1 expression and activity are increased by overexpressing TWIST, ZEB1/2, SLUG, and SNAIL, which results in the development of drug resistance. For example, overexpression of TWIST induced EMT and promoted the growth of multidrug resistance protein 1 (MDR1) in colorectal cancer cells, thereby increasing their resistance to oxaliplatin therapy [46]. Thus, EMT-TFs are expected to provide innovative approaches in the treatment of metastasis and associated drug resistance [54].

MiRNAs are thought to be crucial molecules that connect ABC transporters with EMT in addition to EMT-TFs. MiRNAs are small endogenous long noncoding RNAs [38,55] that are important for controlling the post-transcriptional stage of gene expression and act as negative regulators of mRNA translation and/or stability. They are involved in various biological processes, including cell cycle progression, DNA damage responses and apoptosis, EMT, cell motility, and stemness, through complex transcription factor-miRNA regulatory networks [7,55]. They may also regulate the expression of ABC family genes associated with epithelial-mesenchymal transition (EMT). For example, miR-200c [56] and miR-145 [57]. It is reported to inhibit ABC transporters and also suppress EMT. While another study [58] addressed that the miR-134/487b/655 cluster contributed to the TGF-β1-induced EMT phenomenon and reduced the sensitivity to gefitinib by directly targeting MAGI2, and suppression later caused loss of PTEN stability in lung cancer cells.

## Impression of p53 on epithelial-mesenchymal transition

The p53 gene is a well-studied tumour suppressor gene that is altered or absent in roughly 50 % of human malignancies [59]. p53, also known as the protein product of TP53, has been attributed as a transcription cofactor that acts in response to various stress signals to induce cell cycle arrest, cellular senescence, and apoptosis [42]. Furthermore, the control of cellular metabolism and antioxidative status is identified as a new function of p53 [60]. Recently, p53 has been implicated in the EMT and tumour metastasis regulation through regulating miRNA expression [61]. Moreover, by transcriptionally activating miR-200c, it mediates the features of both EMT and EMT-associated stem cells. Reintroducing miR-200c suppresses genes that regulate EMT and stemness properties and eventually restores the p53-induced mesenchymal and stem cell-like phenotype to the differentiated epithelial cell phenotype. Additionally, loss of p53 is favourably correlated with higher levels of stemness markers and EMT expression, but negatively correlated with miR-200c level and high tumour grade in breast tumours [62].

**Figure 2.**
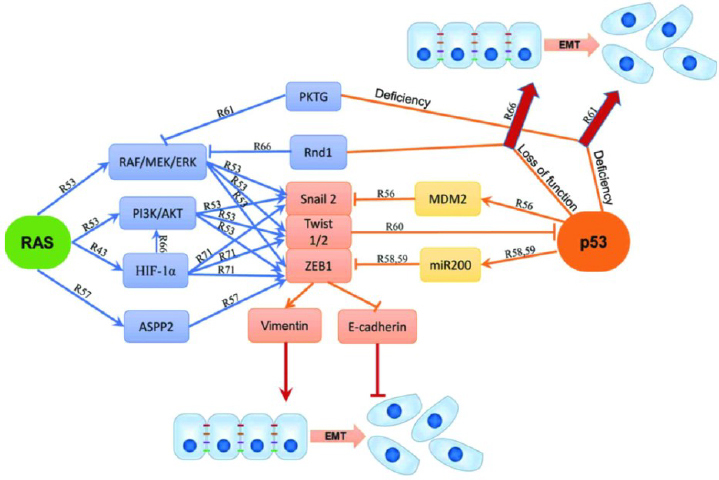
p53 and RAS participate in the regulation of cancer cell EMT [65] (*Creative Commons Attribution CC BY 3.0*)

Previously, researchers mainly focused on p53's role in the cell cycle regulation of apoptosis and genomic stability. Nevertheless, it has been observed recently that loss or inhibition of p53 activity prevents cellular death and contributes to the development of HCC. It has also been revealed that p53 plays a crucial role in controlling HCC cell metastasis and EMT by coordinating the signalling pathways of TGF-β, β-catenin, and PI3K/AKT [63]. Furthermore, mutations involving only one allele of p53 have been reported to impair the wild-type allele p53's normal function, acquire oncogenic properties, or both [64]. Recent evidence has indicated that oncogenic RAS induces EMT in addition to other pathways, such as p53, which is important because EMT is critical for cancer genesis, initiation, and chemoresistance of metastatic tumours. By inhibiting the RAS/PI3K/AKT and RAS/RAF/MEK/ERK pathways, wild-type p53 inhibits human mammary epithelial cells from undergoing RAS-induced EMT and stemness associated with it. Induction of E-cadherin and β-catenin expression is also caused by inhibition of the RAS/RAF/MEK/ERK pathway. Non-small cell lung cancer patients with p53 mutations and low E-cadherin expression have a poor prognosis since RAS-induced EMT is more aggressive. Figure 2 illustrates the precise signal pathways EMT employs, such as p53 and RAS [65].

Furthermore, by blocking the WT p53-miR-200c pathways through dominant-negative effects on WT p53, p53 mutations can promote EMT and the aggressive propensity of tumour cells. Nonetheless, a growing body of research indicates that p53 mutations develop additional carcinogenic roles, such as gain-of-function (GOF), which actively stimulate cells to invade and spread by transactivating or transrepressing a wide range of genes involved in controlling cell adhesion, migration, and proliferation [61].

Furthermore, Kim *et al.* [66] found that mutant p53 directly binds to the promoter of miR-130b, a negative regulator of ZEB1, blocking its transcription, hence exerting oncogenic activities and promoting EMT in endometrial cancer (EC). ZEB1-dependent EMT and cancer cell invasion were induced by the ectopic expression of p53 mutants, which also suppressed the expression of miR-130b. The EMT phenotype was attenuated and ZEB1 expression was decreased because of increased expression of miR-130b following the loss of an endogenous p53 mutant. MiR-130b re-expression also prevented ZEB1 synthesis and mutant p53-induced EMT. Crucially, EC tissues exhibited a significant reduction in miR-130 expression, and patients with higher levels of miR-130b expression lived longer [61]. It has been documented that the higher expression of colorectal cancer CSCs markers is linked to p53 mutants. The p53 has a prominent effect on the maintenance and stability of the genome and stem cells [56]. Therefore, mutations in p53 could be responsible for the aberrant stem cell formation, which are known as CSCs (cancer stem cells). Thus, these results are derived from mutated p53; the mutation may make cancer cells more invasive and aggressive by modifying their behaviour due to the activation of the EMT program. A recent study confirmed that mutant p53 enhances the invasiveness of brain and breast cancer cells by activating YAP/TAZ signalling, as these signals regulate the EMT process. This established connection between EMT and CSCs development is due to the p53 mutation, which can be considered a milestone in the molecular events that contribute to the oncogenic aggressiveness of CSCs [66,67].

## Therapeutic molecules in epithelial-mesenchymal transition regulation

A comprehensive compilation of EMT modulators of different sources from plants, animals, algae, and bacteria is shown in Table 1.

**Table 1.** Role of plants, animals, and other therapeutic molecules in EMT regulation

Role of plant-based therapeutic molecules in EMT regulation				
Based	Source	Chemical constituents	Marker	Reference
Terrestrial	*Atractylodes lancea* DC.	Sesquiterpenoids	AT-III	[70]
Terrestrial	*Dendrobium officinale* Kimura & Migo	Flavonoid glycoside	Vicenin II	[74]
Terrestrial	*Panax ginseng* C.A. Mey.	Saponins	Rg3	[75,76]
Terrestrial	*Platycodon grandiflorus A*. DC.	Saponins	Platycodin D	[86]
Marine	*Aquimarina* sp. MC085	Lactone	Caprolactin C	[68]
Marine	*Dictyota dichotoma (Desfontaines) J. V. Lamouroux*	Polysaccharide	Laminaran sulfate	[90]
Marine	*Bangia fuscopurpurea* (Dillwyn) Lyngbye	Polysaccharide	BFP-3	[91]
Marine	*Posidonia oceanica* (L.) Delile	Polyphenol	Catechin, epicatechi, chlorogenic acid	[94]
Role of animal-based therapeutic molecules in EMT regulation				
Terrestrial	*Appis mellifera* (Bee venom)	Peptides, enzymes, and proteins	Apamin, melittin phospholipase A2	[95]
Terrestrial	Taiwan cobra (*Naja naja, Naja naja atra*) (Snake venom)	Protein, polypeptides	Cystatins, cystatin M and cystatin C; cardiotoxin III	[106]
Marine	*Bugala neritina*	Macrolide	Bryostatin 1	[110]
Marine	*Sinularia flexibilis*	Diterpene lactone	Sinulariolide	[112]
Marine	Haliclona (sponge)	Alkaloid	Manzamines	[116]
Marine	*Halichondria okadai* (sponge)	Macrolide	Halichondrin B and eribulin	[119]
Marine	*Caenorhabditis elegans*	Pyrimidine derivatives	Biemamides	[121]
Role of others therapeutic molecules in EMT regulation				
Algae	*Fucus vesiculosus*, *Laminaria digitata* and *Ascophyllum nodosum*	Polysaccharide	Fucoidan	[123]
Bacteria	*Streptomyces hygroscopicus*	Macrocyclic lactone	Rapamycin	[126]

### Role of plant-based therapeutic molecules in EMT regulation

#### A. *Atractylodes lancea*

Rhizome of the *Atractylodes macrocephala Koidz* plant constitutes the principal component of Baizhu, a traditional Chinese medicine that is commonly employed for the treatment of gastrointestinal disorders [68,69]. Numerous prescriptions for traditional medicine based on Baizhu and the similar preparation Cangzhu are utilized as Qi boosters in China, Korea, and Japan. Aractylenolides, a minor class of sesquiterpenoids endowed with antioxidant and anti-inflammatory properties, are present in these preparations. Furthermore, Atractylenolides I, II, and III exhibit noteworthy anticancer characteristics. An observed impediment to cell differentiation was identified in the presence of AT-III, specifically through the inhibition of the EMT induced by TGF-β1 [70]. Additionally, cell migration could be impeded by AT-I and AT-II [71]. AT-I's anti-metastatic effect may be partially attributed to its ability to inhibit the transfer of the pro-metastatic microRNA miR-200c into cells via extracellular vesicles, thereby suppressing the activity of this microRNA [72]. AT-III inhibits the TGF-1-induced differentiation of MDA-MB-468 epithelial cancer cells into mesenchymal cells by suppressing the expression of vimentin and N-cadherin, which are mesenchymal markers acquired during EMT [70].

#### B. *Dendrobium officinale* Kimura & Migo

Vicenin II will be among the most significant constituents accountable for the anti-metastasis effect of *Dendrobium officinale* Kimura & Migo. TGF-β1 is responsible for EMT initiation. By inhibiting TGF-β1-induced EMT phenotypes in lung adenocarcinoma A549 and H1299 cells, TGF-β1 stimulated spindle-shaped alterations, enhanced migration and invasion, and either increased or decreased the relative expression of biomarkers associated with EMT [73]. The induction of EMT by TGF-β1 was impeded by Vicenin II through the inhibition of the PI3K/Akt/mTOR and TGF-β/Smad signalling pathways. It is the first time that the molecular mechanisms underlying the anti-metastatic effects of Vicenin II have been elucidated. Moreover, by interfering with TGF-ββ1-induced EMT, Vicenin II may serve as a promising repressor against the metastasis of lung adenocarcinoma [74].

#### C. *Panax ginseng* C.A. Mey.

The numerous health benefits of *Panax ginseng* C.A. Mey. (Araliaceae) root have long been well known. These come from the proven fact that its components control signal pathways that are involved in inflammation, oxidative stress, angiogenesis, and cancer propagation [75,76]. Recently, it has been said that ginseng extract is a good way to treat cancer because it contains anticancer substances such as Rh2, Rg3 and Rg5 saponins. Ginsenosides are active pharmaceutical ingredients that are taken from ginseng, which is a traditional Chinese medicine. Forty different kinds of ginsenoside compounds have been found so far. Of these, Rg3 is getting increasing attention. Rg3 has more than one effect on tumours. Rg3 stops colorectal cancer cells from growing by blocking the Wnt/β-catenin pathway [77], helps ovarian cancer cells die by blocking the PI3K/AKT pathway [78], stops esophageal cancer cells from making new blood vessels when oxygen levels drop [79], and stops EMT in ovarian cancer by decreasing HIF-1α [80]. Lung cancer EMT and invasion can't happen because Rg3 stops FUT4 from turning off EGFR and stops MAPK and NF-κB signal pathways. It's speculated that Rg3 could be an advantageous medication for treating lung cancer [81].

#### D. *Platycodon grandiflorus* A. DC

The primary saponin extracted from Platycodonis radix, a two to three year old root of *Platycodon grandiflorus* A.DC., is Platycodin D [82,83]. Notably, emerging evidence suggests that Platycodin D may possess antitumor properties against various malignancies, including but not limited to lung cancer [82], gastric cancer [84], and hepatocellular carcinoma [85]. By regulating the LncRNA-XIST/miR-335 axis, PD inhibits the *in vitro* and *in vivo* development of bladder cancer. In bladder cancer cells, Platycodin D promoted cell apoptosis and mechanistically suppressed malignant phenotypes, including invasion, migration, proliferation, and EMT, in a dose and time-dependent fashion. It was unique to boost the antitumor effects of Platycodin D in bladder cancer *in vitro* and *in vivo* by targeting the LncRNA-XIST/miR-335 axis [86].

#### E. Caprolactin C

Caprolactin extracted from the aqueous extract of the *Aquimarina* genus exhibits a potent inhibitory effect on EMT signalling. A recent study conducted by Kim *et al.* [87] illustrated the effect of caprolactin C on lung cancer cells and found that cells treated with caprolactin C showed the inhibition of the phosphorylation of SMAD2/3 and inhibited the EMT signalling pathways via inhibition of β-catenin, N-cadherin. However, further study is needed to understand the in-depth function of caprolactin, as it may be a potential antimetastatic agent due to its inhibition of TGF-induced EMT.

#### F. Laminaran sulphate

By suppressing MMP-2 and MMP-9 activity in HCT116 cells (human colorectal adenocarcinoma), SK-MEL-5 cells (malignant melanoma), and MDA-MB-231 cells (breast adenocarcinoma), laminaran sulphate, which is extracted from the brown alga *Fucus evanescens*, was shown to be an effective anti-migratory drug [88,89]. In another pioneering research, Malyarenko *et al.* [90] showed that sulphated laminaran, extracted from the brown alga *Dictyota dichotoma* (Desfontaines) J.V. Lamouroux, may modulate the function of MMP-2 and MMP-9, thereby enhancing the effect of X-rays and stopping the migration of SK-MEL-28 melanoma cells. This study offers a new method of fusion of cancer radiation treatment.

#### G. BFP-3

Wu *et al.* [91] *a*lso found that a new water-soluble polysaccharide (BFP-3) isolated from the red alga *Bangia fuscopurpurea* (Dillwyn) Lyngbye stopped A2780 ovarian cancer cells from spreading and migrating. The ability of BFP-3 to inhibit migration was likely due to mechanisms that induce apoptotic and autophagic cell death through a pathway that depends on mitochondria.

#### H. Catechin, epicatechin, chlorogenic acid

The hydroalcoholic extract of *Posidonia oceanica* (L.) Delile contains high levels of the phenolic compounds gallic acid, chlorogenic acid, epicatechin, and ferulic acid. It has been shown to prevent the migration of human fibrosarcoma HT1080 cells [92] and human neuroblastoma SHSY5Y cells [93] by affecting matrix metalloproteinases and triggering autophagy. Nanoformulations can maximize absorption and migrastatic bioactivity by utilizing the carrier role of polyphenol-impregnated phytocomplexes [94].

### Role of animal-based therapeutic molecules in epithelial-mesenchymal transition regulation

#### A. Bee venom

As a form of defensive venom, bee venom (BV) is produced and retained in the abdominal poison sac by the venom glands (*Apis mellifera*) [95]. The substance in question comprises peptides, enzymes, and smaller proteins, including apamin, melittin (MEL), and phospholipase A2 (PLA2), among others, which consist of amines, carbohydrates, and minerals [95,96]. Through these active constituents, BV elicits a range of varied pharmacological responses. Several evaluations have examined the pharmacological advancements of BV, with a particular focus on its antitumor properties [97,98].

By exhibiting multi-pathway and multi-target lasting impacts on cells *in vitro* & *in vivo*, bee venom is crucial in regulating the cell proliferation, invasion, migration, suppression of EMT, apoptosis induction and autophagy in cancer types, such as lung, breast, cervical and numerous other types of cancer. BV exerted its effects through the following mechanisms: expression downregulation of EGFR, MITF, Erα, PARP, VEGF, Bcl-2, Caspase-7, Bcl-xL, MMP-1, Caspase-9, PTEN, p21, p53, p27, Rb, Bax, and 15-lipoxygenase-1; and Cyclin A, NF-κB, Cyclin D1, Cyclin B, HIF-1α, and Rac1. By downregulating the expression of p-mTOR, p-EGFR, p-Akt, p38, JNK, p-p38, p-JNK, ERK, p-PI3K, Akt, and p-HER2, BV impeded the mTOR, PI3K/Akt, and MAPK signalling pathways, as well as the mitotic signalling pathway. Adverse effects included poor and impaired carcinoma cell viability, alleviated migration and invasion activities, which enhanced the death of cells. The apoptotic pathway of cancer cells' mitochondria was stimulated by BV through an upregulation of apoptosis signalling molecules, including Fas and Caspase-9. Conversely, it suppressed EMT in cancer cells by upregulating E-cadherin and downregulating the expression of vimentin, ZEB2, and Slug [99].

#### B. Snake venom

Toxins extracted from snake venoms have the potential to serve as a natural reservoir of molecular scaffolds for the development of agents that inhibit the migration and invasion of cancer cells [100]. By reversing EMT induced by EGF and HGF (hepatocyte growth factor), cardiotoxin III (CTX-III), a membrane toxin derived from the venom of the Taiwan cobra (*Naja naja*) [101], suppresses the migration of cancer cells. EGF-induced EMT in breast cancer cells is inhibited by CTX-III, which also activates PI3K/Akt and ERK1/2 and decreases EGFR phosphorylation. It increases E-cadherin levels and decreases MMP-9 and the mesenchymal markers vimentin and N-cadherin, thereby inhibiting EGF-induced invasion and migration. An analogous impact of CTX-III on the migration and invasion of breast cancer cells stimulated by HGF has been documented [102-104].

A snake venom cystatin (Sv-cystatin) with a shorter sequence than other type-2 cystatins, including cystatin M and cystatin C, has been isolated from the venom of *Naja naja atra*. In MHCC97H liver cancer cells, the inhibitory effects of this snake toxin on invasion and metastasis have been described [105]. These effects are mediated by a reduction in EMT markers. Sv-cystatin reduces the levels of MMP-2, MMP-9, and cathepsin B activity, while increasing E-cadherin and diminishing the EMT proteins TWIST and N-cadherin [106].

#### C. Bryostatin 1

Bryostatin 1 is found in the marine invertebrate Bugula neritina [107] and exhibits significant potency in inhibiting various cancer-regulated pathways [108]. The availability of bryostatin 1 was found to be higher in the B. neritina larvae as compared with the adult ones, which suggests the significant importance of this compound in the larval stage [109]. During cancer progression, the EMT pathways are initiated by cancer cells, leading to a higher metastasis rate and increased cancer invasion. Studies reported the major involvement of the PKC signalling pathways and EMT signalling, which results in higher association in the PKC phosphorylation, inducing EMT signalling. Bryostatin 1 acts as an antagonist to the EMT pathways and shows a significant inhibition of the PKC pathways [110]. Previous *in vitro* studies have shown a higher rate of inhibition of PKC pathways when treated with cancerous cells using bryostatin 1 [110], but when implemented in human studies, it showed no significant effects [111].

#### D. Sinulariolide

Sinulariolide is a biologically active compound derived from the marine soft coral species Sinularia flexibilis, exhibiting potent anticancer activity. During the proliferating stage of cancer, the cancerous cells undergo various pathways upregulation, such as Fak/PI3K/AKT/mTOR and MAPKs. The primary consideration in cancer research is to inhibit the sequential pathways supporting cancer growth. Various studies show the inhibition of the proliferation and metastasis properties of cancer cells when treated with sinulariolide through downregulation of PI3K/mTOR and p38MAPK pathways in bladder and hepatic cancer [112,113]. One recent study showed the potential effect of sinulariolide on cancer cells and the results showed a significant reduction in the expression of various proteins, such as MMP9, p39MAPK, mTOR, ERK, and inhibited the EMT process in cancerous cells [114].

#### E. Manzamines

Manzamines are bioactive marine-derived alkaloids extracted from the sponge called *Haliclona*, and demonstrate a promising effect on cancer cell growth inhibition [115]. Various studies conducted on cancer cells using Manzamine show the positive effect on cancer cell proliferation and apoptosis. One study showed the effect of Manzamine on colorectal cancer cells. When cancerous cells were treated with Manzamine, it induced the inhibition of cyclin-dependent kinase via p53 pathways. A bioinformatics study demonstrated the complete abolishment of EMT signalling via suppression of TWIST and SNAIL, along with suppression of metastasis of cancer [116]. Another study showed the antiproliferation effect of Manzamine on cervical cancer and its results demonstrated the increased antiproliferation activity on cancerous cells in a dose-dependent manner with Manzamine [117].

#### F. Halichondrin B and eribulin

Halichondrian B was obtained from the *Halichondria okadai* sponge. Halichondrin B showed great cytotoxic activity against the solid tumours [118]. The mechanism of halichondrin B illustrates that it inhibits tubulin-dependent guanosine triphosphate hydrolysis and tubulin polymerization [119]. The problem associated with Halichondrin B is low yield, which was overcome in 1992 by the Kishi lab. They developed many synthetic analogues, and the best studied compound is called eribulin. Mechanistic studies depict that eribulin lowers the expression of angiogenesis genes and the signalling pathways such as Wnt, Notch and EMT [120].

#### G. Biemamides

Biemamides are a natural marine product obtained by screening the library of natural marine products and revealed five biemamides A-E [121]. Biemamides show potent anticancer activity in the context of cancer size reduction and inhibition of TGF-β pathways. A recent study revealed the action of biemamides *in vitro* using the NMuMG cell line. In this study, the author found that the cells treated with biemamides regulated the downregulation of the TGF-β pathways, which is the main player in cancer metastasis [122].

### Role of bacteria and algae-based therapeutic molecules in epithelial-mesenchymal transition regulation

#### A. Fucoidan

Fucoidan is a naturally occurring sulphated polysaccharide compound found in the various species of brown algae such as *Fucus vesiculosus, Laminaria digitata* and *Ascophyllum nodosum*. The role of fucoidan as an anticancer therapy has developed over the last 10 years because of its involvement in the various multistep processes in cancer inhibition through modulation of cell cycle regulation, autophagy, apoptosis, metabolism and PI3K-AKT-mTOR pathways. Various studies supported the role of Fucoidan in the apoptosis of carcinogenic cells and its modulation in the EMT pathways [123]. A previous study reported the effect of fucoidan on the reversible mechanism of EMT signalling pathways, which is induced by the TGF-β receptor expression [124]. The effectiveness of a 50 % reduction in cell proliferation with 100 μg fucoidan in A549 lung cells [125].

#### B. Rapamycin

Rapamycin, also known as sirolimus, is extracted from the surface bacterium *Streptomyces hygroscopicus* and found on Easter Island [126]. Rapamycin was initially developed as an antifungal agent [127] but later found to exhibit antiproliferative activity due to the inhibition of the mTOR pathways [128]. A previous study reported the inhibition of EMT signalling through suppression of the mTOR pathway, when brain tumour cells were treated with rapamycin in a dose-dependent manner [129]. Another study also reported the potential effect of rapamycin and demonstrated the mechanism of pathway inhibition of the mTOR pathways in oncogenesis [130]

## Alternative therapeutic approaches for EMT treatment

Several alternative therapeutic strategies have been developed for the inhibition and reversal of EMT in cancer. These strategies are aimed at interrupting the major molecular mechanism that is responsible for the EMT processes, such as converting mesenchymal-like cancer cells to an epithelial phenotype or inhibiting the gain of EMT-related characteristics such as increased invasiveness, metastatic capability, and resistance to drugs.

The RNA interference (RNAi) technology, based on small interfering RNAs (siRNAs) and microRNAs (miRNAs) can become a potential tool for gene silencing, which is involved in EMT. It has been documented that the EMT drives transcription factors like SNAI1, ZEB1, TWIST1 and SNAI2 (Slug) that can be knocked down by siRNAs. As a result, reverse mesenchymal traits and restore epithelial features have been observed in several cancers. Moreover, miRNAs such as miR-200 family and miR-205 are established repressors of EMT. Restoration of these miRNAs within cancer cells can suppress migration and invasion through direct targeting of ZEB1/2 and other EMT markers [131-133].

The epigenetic regulation has an important role in maintaining the EMT state by altering the chromatin structure and gene accessibility. By altering acetylation profiles, histone deacetylase inhibitors, such as vorinostat and trichostatin A, can induce the re-expression of epithelial markers, including E-cadherin. Decitabine, a DNA Methyltransferase inhibitor, can demethylate the CDH1 promoter (E-cadherin gene), thereby reversing silencing and preventing EMT. The potential of epigenetic therapies to sensitize tumours to chemotherapy and lower their ability to metastasize to other sites in the body and makes it applicable to use in combination with conventional therapies [134,136]. TGF-β inhibitors can inhibit canonical SMAD signalling and diminish EMT induction in pancreatic, breast and lung cancers [134,135]. The LGK974, a Wnt/β-catenin pathway inhibitor, can inhibit nuclear translocation of β-catenin and repress mesenchymal gene expression [138]. EGFR inhibitors like erlotinib and gefitinib, in combination with anti-inflammatory drugs, can inhibit proliferation and affect EMT [139]. The γ-secretase is a Notch inhibitor that can inhibit glioblastoma and colorectal cancer EMT by inhibiting Notch-1 signalling [140].

CRISPR/Cas9 technology presents a strong and targeted approach to knock out or correct genes implicated in EMT. CRISPR has been utilized to knock out ZEB1, SNAI1, and TWIST1 in research cancer models, resulting in decreased invasion and metastasis. Gene editing also permits the targeting of non-coding RNAs and enhancer factors controlling EMT transcriptionally [141,142]. Nanotechnology and targeted drug delivery, including lipid nanoparticles, polymeric micelles, and exosomes, have been utilized to deliver miRNAs, siRNAs, or small-molecule inhibitors directly to tumour cells. Nanoparticles delivering miR-200c were shown to reverse EMT and suppress metastasis in triple-negative breast cancer models [143,144]. Furthermore, recent findings suggest that EMT plays a role in immune evasion mechanisms. Therefore, the combination of immunotherapy with EMT-targeted interventions may have synergistic effects. CSF1R, TGF-β, or IDO1-blocking therapies can modulate the tumour microenvironment and suppress EMT-fostering, immune-suppressive signals [145].

## Conclusion

In conclusion, understanding the regulatory intricacies of EMT and MET is crucial for tackling cancer progression and drug resistance. This review emphasizes promising natural compounds, such as those from *Atractylodes lancea*, *Dendrobium officinale* Kimura & Migo, *Panax ginseng* C.A. Mey., *Platycodon grandiflorus* A. DC., bee venom, snake venom, and marine sources, with anti-metastatic potential by regulating EMT pathways. Additionally, recognizing the role of MET in sustaining distant metastasis and the impact of p53 on EMT underlines the complexity of these regulatory networks. The diverse therapeutic molecules discussed offer prospects for targeted strategies. Future research should focus on deciphering molecular intricacies, clinical validation, and developing combination therapies for more effective cancer metastasis treatment. Advancing our understanding of EMT and MET opens avenues for novel approaches in the fight against cancer metastasis.
